# Corrosion and biocompatibility behaviours of microarc oxidation/phytic acid coated magnesium alloy clips for use in cholecystectomy in a rabbit model

**DOI:** 10.1039/d0ra09275d

**Published:** 2021-06-10

**Authors:** Qiuxia Zheng, Zongbin Sun, Zhanhui Wang, Tinghe Duan, Kai Xu, Mengmeng Cai, Bi Wang

**Affiliations:** Department of Surgery, Luoyang Central Hospital Affiliated to Zhengzhou University 288 Zhongzhou Road Luoyang 471000 China zhanhuiwang868@163.com +86 379 6389 2095 +86 379 6389 2095

## Abstract

With the popularisation of laparoscopic cholecystectomy, ligation clips have been commonly used for ligating the cystic duct and cystic artery. However, non-degradable clips remain in the body long-term, which significantly increases the risk of the clip becoming detached. Thus, magnesium alloys have attracted tremendous attention owing to their biodegradability and good biocompatibility. However, the poor corrosion resistance hinders the clinical application of magnesium alloys with microarc oxidation/phytic acid (MAO/PA) composite coatings as protective coatings. Here, these alloys were used to hinder the rapid material degradation in aqueous solution. Electrochemical tests were conducted to evaluate the *in vivo* degradation behaviour in simulated body fluid (SBF) for Mg–Zn–Y–Nd alloys, and scanning electron microscopy (SEM) was used to observe the micromorphology of *in vivo* clip degradation. Cell toxicity, cell adhesion, and flow cytometry were performed *in vitro* to detect cytocompatibility. Biochemical detection of serum magnesium, serum creatinine (CREA), blood urea nitrogen (BUN), alanine transaminase (ALT), and alanine aminotransferase (AST), and haematoxylin–eosin (HE) staining of the heart, liver, and kidney tissues *in vivo* was conducted to determine the biocompatibility properties after surgery. Electrochemical measurements and SEM images revealed that the MAO/PA-coated magnesium alloy delayed corrosion in SBF. The apoptosis rate increased slightly with increased extract concentration. Nevertheless, MAO/PA-coated magnesium alloys still exhibited good cytocompatibility. No obvious abnormality was observed in the blood biochemical test or HE staining. Thus, MAO/PA-coated magnesium alloys exhibit better corrosion than bare magnesium. In addition, Mg–Zn–Y–Nd and MAO/PA-coated magnesium alloys exhibited no cytotoxicity, good adhesion, and biosafety.

## Abstract 2

Mg alloys with microarc oxidation/phytic acid composite coatings were tested as degradable ligation clips for ligating the cystic duct and cystic artery.

## Graphical abstract

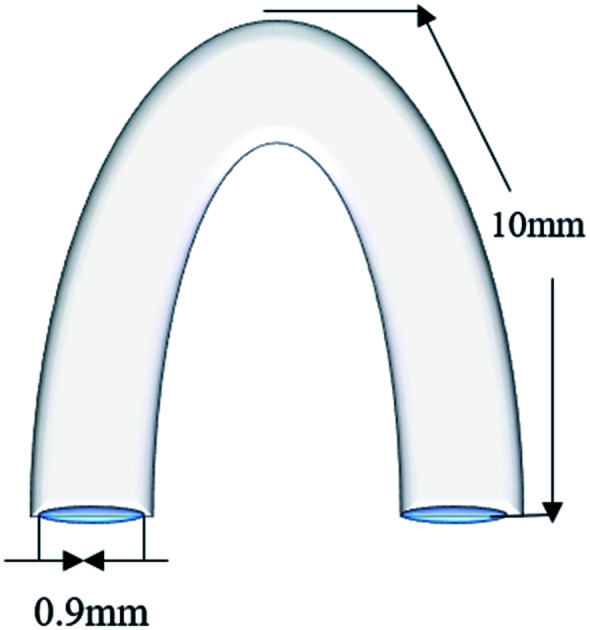

## Introduction

Laparoscopic cholecystectomy (LC) is currently the first choice for treating symptomatic cholelithiasis.^1^ Hem-o-lok and titanium clips, which are non-degradable materials, are extensively used for ligation during the procedure.^2,3^ However, permanent metal implants inevitably cause side effects, including thrombosis, permanent physical stimulation, and local response to chronic inflammation.^4,5^ In addition, titanium clips can cause a local inflammatory response in the body,^6^ which can form metallic artefacts in imaging examination.^7^ Consequently, magnesium alloys have been extensively considered as promising alternatives owing to their good biocompatibility and biodegradability.^8^ Recently, magnesium alloys have been extensively investigated as implants for orthopaedic and cardiovascular applications, and in oral and maxillofacial surgery.^7,9,10^ One reason is that magnesium alloys and human bone tissue exhibit a similar elastic modulus, and magnesium ions can induce the activation of osteoblasts.^11,12^ Another reason is that magnesium ions play an effective role in preventing late thrombosis.^13^ In addition, Mg–Zn–Y–Nd alloys have been investigated as biomaterials for intestinal, vascular, and oesophageal stents, which indicates their potential feasibility for clinical applications.^14–16^

However, the major limitation of magnesium alloys is their poor corrosion resistance.^17^ Thus, the mechanical strength of the magnesium alloy biomaterials decreases after implantation; further, the local implant position is in an alkaline environment. If the large amount of hydrogen released is not absorbed in time, emphysema will occur in the loose tissue.^18^ Accordingly, the low corrosion resistance of magnesium alloy materials limits their clinical applications. Hence, numerous approaches, such as surface modification of the coatings and adequate alloying elements, have been developed to address these limitations, such as surface modification of the coatings and selection of adequate alloying elements.^17^ Magnesium alloys significantly reduce the degradation rate by surface protective coatings.^18^ In our previous study, we used a microarc oxidation (MAO) treatment as an economic surface treatment method to improve the corrosion resistance,^19^ because it relates to the high-pressure plasma-assisted anodic oxidation process and promotes the formation of highly adhesive ceramic oxide coatings. Therefore, the mechanical strength and excellent corrosion resistance of magnesium alloys have been improved.^20^ Nevertheless, since the porous surface of the MAO causes heterogeneous degradation, it can only improve the early corrosion resistance; in the later stage after implantation, the degradation rate was accelerated.^21^ Phytic acid (PA) is a natural, non-toxic organic acid that exists in the cytoplasm and nucleus of eukaryotic cells and has a strong chelating capacity with metal ions such as magnesium and zinc ions.^22,23^ In addition, as a surface conversion film, PA has a good protective function on the surface of magnesium alloys.^24^ Assuming that the pores of MAO form the first layer on the surface of the magnesium alloy, PA covered the MAO to form the outermost layer, which is the fabrication process for MAO/PA composite coatings, which can achieve a better corrosion resistance than single coatings.

Thus, MAO/PA composite coatings were synthesised on the Mg–Zn–Y–Nd alloy. The corrosion resistance was investigated by electrochemical tests and scanning electron microscopy (SEM) images, and the effects of biocompatibility were detected by *in vitro* cell experiments and tests. Through the analysis of corrosion and biocompatibility performances, this study aims to investigate the feasibility of dual-coated magnesium alloy clips for use in cholecystectomy in a rabbit model. It provides theoretical support for the clinical application of this type of biodegradable magnesium alloy clip.

## Materials and methods

### Processing of the materials

The extruded Mg–2.0% Zn–0.5% Y–0.5% Nd alloy was prepared at Zhengzhou University and contained master alloys of induction heating high-purity Mg, high-purity Zn, Mg–25Y (wt%) (99.99% purity), and Mg–25Nd (wt%) (99.97% purity), which were mixed thoroughly and melted at 740 °C under a CO_2_/SF_6_ atmosphere (volume fraction, 3000 : 1) in an electronic resistance furnace.^25^ Then, the Mg–Zn–Y–Nd alloy was extruded into rods and cut into round disks (*Φ* 8 × H3 mm).

**Fig. 1:**
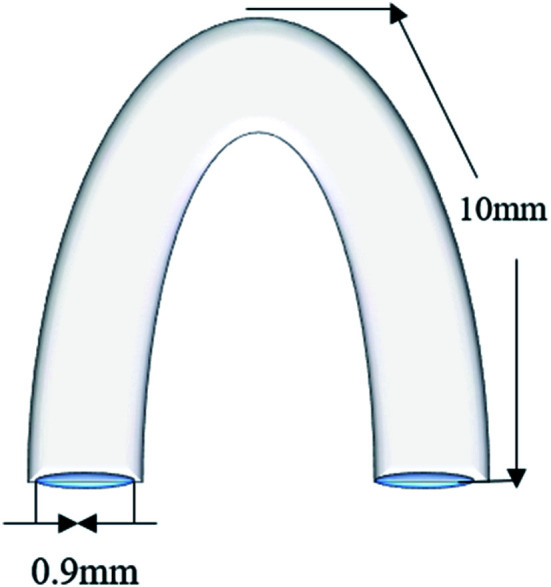
Schematic diagram of clips.

Thereafter, the samples were mechanically ground to 2000# with grit SiC paper and cleaned sequentially in acetone, ethanol, and deionised (DI) water. After drying, some samples underwent electrochemical tests, and others were sterilised with ultraviolet rays for 1 h and prepared for *in vitro* cell experiments. A number of clips (cross-section diameter: 0.9 mm, side length: 10 mm) were fabricated (Fig. 1) using a drawing machine, and V-shaped clips were formed by weaving. Eventually, all clips were treated by annealing to obtain better mechanical properties in the animal experiments.

### Coating preparation

The prepared specimens were anodised using an MAO machine (YS9000D-300-40), after DI water was added to trisodium phosphate, sodium hydroxide, and glycerol and the MAO electrolyte was prepared. The magnesium alloy samples underwent MAO treatment (20 min). A stainless steel sheet was used as the negative electrode with a frequency of 800 Hz, a voltage of 260 V, and a duty cycle of 15%. The specimens were subsequently washed with DI water and air-dried. The phytic acid solution was adjusted to pH 5 with sodium hydroxide to a concentration of 7.5 g L^−1^, and the magnesium alloy coating with MAO samples was placed in the PA solution in a constant-temperature water bath at 40 °C, rinsed with DI water again, and dried in cold air. The Mg–Zn–Y–Nd alloy coated with MAO/PA was set as the experimental group, and the uncoated samples were used as the control group.

### Detection of corrosion properties

#### Electrochemical test

An RST5200 electrochemical workstation was used to investigate the corrosion performance of the MAO/PA-coated magnesium alloy in SBF. The traditional three-electrode system was used for the analysis; the reference, counter, and working electrodes were a saturated calomel electrode, a platinum plate, and magnesium alloy round disks, respectively. Then, potentiodynamic polarisation was demonstrated by the electrochemical workstation.

#### SEM observation

The clips of different treatment groups were removed after implantation at three weeks, washed with DI water, then air-dried. SEM was used to observe the surface corrosion morphology.

### Cytocompatibility *in vitro*

#### Cell cultures and preparation of extracts

Human fibroblasts, purchased from Shanghai Zhong Qiao Xinzhou Biotechnology Co., Ltd. Shanghai, China, were used for the cytocompatibility tests. The fibroblasts were cultured in Dulbecco's modified Eagle's medium (DMEM) with high glucose in a 5% CO_2_ incubator with 95% relative humidity at 37 °C, containing 20% fetal bovine serum (FBS) and 1% penicillin/streptomycin antibiotics. The sterilised round disks were placed into 24-well plates, according to ISO 10993-5:1999,^26^ and the ratio of the amount of DMEM medium to the surface area of the magnesium alloy sample was 1.25 mL : 1 cm^2^. The specimens were then placed into the cell incubator and cultured for 24 h at 37 °C in a 5% CO_2_ incubator. The extracts were collected, centrifuged, and subsequently diluted to concentrations of 25%, 75%, and 100% with DMEM. The DMEM medium was used as the negative control and 0.64% phenol DMEM medium was used as the positive control.

#### CCK-8 assay

CCK-8 assay was used to assess the viability and growth conditions, and the human fibroblast suspension was added to a cell concentration of 1 × 10^5^ cells per mL, which was seeded in 96-well plates and cultured for 24 h for attachment. Then, the cell medium was replaced by 25%, 75%, 100% concentrations of MAO/PA coating magnesium alloy and bare group extracts. After adding 10 μL of the CCK-8 reagent and incubating for 2 h, the cell morphology and quality were observed using optical microscopy, and the optical density value (OD) at 450 nm was calculated using a microplate reader. The cell survival rate was calculated using the following formula:Relative cell survival rate = (average absorbance value of experimental group − average absorbance value of positive control group)/(average absorbance value of negative control group − average absorbance value of positive control group).

#### Cell adhesion test

The early cell adhesion behaviours of MAO/PA-coated and uncoated magnesium alloys were investigated by fluorescence microscopy. A cell suspension with a density of 5 × 10^4^ cells per mL was seeded on the surface of a sterilised magnesium alloy sample. After the cells were completely precipitated on the magnesium alloys, they were added to the required medium and cultured for 24 h. Subsequently, human fibroblasts were washed with PBS and fixed with 95% alcohol for 30 min. The cells were stained with 0.01% acridine orange. After 10 min, the morphology, quantity, and growth status of the cells were observed by fluorescence microscopy.

#### Flow cytometry of apoptotic cells

An Annexin V-FITC apoptosis detection kit (Beyotime, Shanghai) was used to evaluate the effect of magnesium alloy extract concentrations of 0%, 25%, 75%, and 100% on human fibroblast apoptosis. Human fibroblasts were plated at a density of 2 × 10^5^ cells per mL in 24-well plates and cultured with DMEM after 24 h. The culture medium was aspirated and rinsed softly three times with PBS, added to different extract concentrations, then cultured for 24 h. The cells were then digested and centrifuged. They were subsequently resuspended in the 195 μL V-FITC Annexin binding solution, thoroughly mixed, then Annexin V-FITC solution (5 μL) was added. After incubation at 25 °C for 10–20 min, the results were analysed by flow cytometry. Unstained cells were defined as live cells, and cells stained with Annexin V only were classified as early apoptotic cells.

### *In vivo* test

#### Animal surgery

Animal experiments were approved by the Animal Research Committee of Zhengzhou University, and the surgical procedure complied with the guidelines for animal care and use of the National Institutes of Health and the American Heart Association. Thirty adult New Zealand rabbits (average weight: ∼2.0–2.5 kg) were used in this experiment, and were randomly divided into two groups: MAO/PA coating group and uncoated group; each group was five. The rabbits were infused with antibiotics and analgesics before the operation and were operated under general anaesthesia. The abdominal rabbit hair was shaved and disinfected with complex iodine. The site of the linea alba as a surgical incision was exposed to the triangle of Calot, and the locations of the cystic duct and cystic artery were identified. The cystic duct was ligated using MAO/PA coated or uncoated magnesium alloy clips. The cystic artery was ligated with silk thread, the gallbladder was removed, the abdomen was closed, and the incision was disinfected. After the operation, the rabbits were treated with analgesics after recovery, and the appropriate amount of normal saline and glucose solution was infused according to the amount of bleeding. The activity of the rabbits was then closely observed.

#### Biochemical tests

Serum alanine transaminase (ALT), alanine aminotransferase (AST), creatinine (CREA), and blood urea nitrogen (BUN) levels were detected after cholecystectomy. Blood samples (2 mL) were collected through the ear vein after surgical operation at 3, 7, 14, and 21 d, and blood samples were measured using a blood biochemistry analyser.

#### HE staining

The rabbits were euthanised three weeks after cholecystectomy, and the heart, liver, and kidneys were removed in 10% formaldehyde solution. Subsequently, the tissues were embedded in paraffin and cut into thin sections (thickness: ∼5 μm), processed and stained with haematoxylin and eosin, and the pathological sections were assessed using optical microscopy.

## Results

### Corrosion resistance results

#### *In vitro* electrochemical measurements

**Fig. 2:**
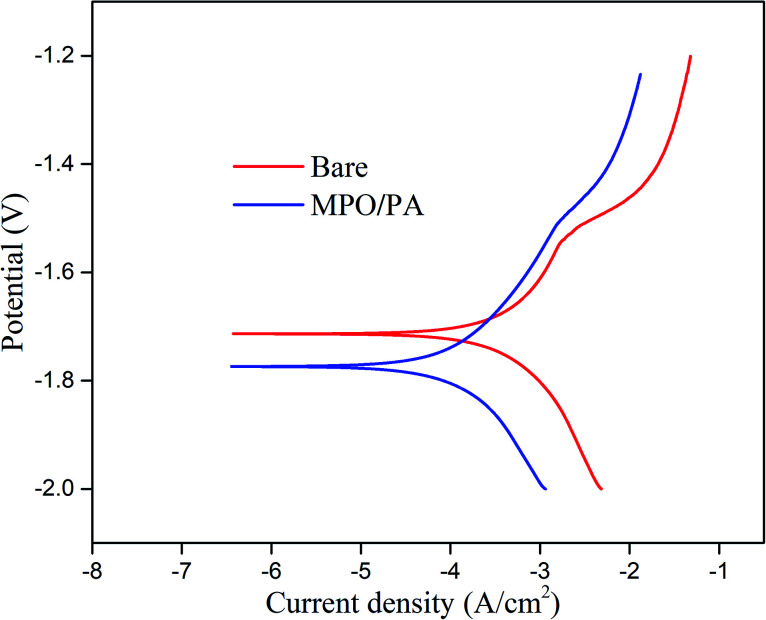
Polarisation curves for MAO/PA coating and bare samples.

The polarisation curves of the MAO/PA-coated and bare Mg–Zn–Y–Nd alloys are shown in Fig. 2. The corrosion potential (*E*_corr_) and corrosion current density (*i*_corr_) were calculated by the Tafel method. The parameters of the polarisation curves are listed in Table 1, which shows that the *i*_corr_ of the MAO/PA-coated samples is one order of magnitude lower than that of the uncoated samples, indicating that the corrosion resistance of the MAO/PA-coated samples was superior to that of the bare samples.

**Table 1:** Fitted parameters of the polarisation curves for MAO/PA-coated and bare samples

Sample	*E*_corr_ (V)	*i*_corr_ (A cm^−2^)
Bare sample	−1.71	1.2 × 10^−4^
MAO/PA-coated Mg alloy	−1.77	3.5 × 10^−5^

#### SEM observation

**Fig. 3:**
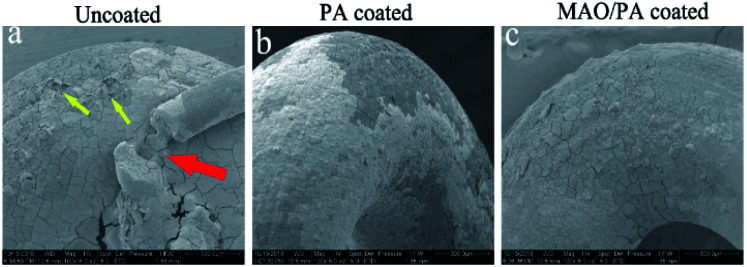
Surface corrosion morphologies of clips implanting the rabbits at three weeks: (a) uncoated; (b) PA coated; (c) MAO/PA coated. Red arrow shows the corrosion products.Yellow arrows show the small corrosion pits (100×).

Fig. 3 depicts the surface corrosion morphologies of the clips implanted in the rabbits at three weeks. In Fig. 3a, some corrosion products fell off (red arrow) and several small corrosion pits occurred in the uncoated group (yellow arrows), mainly due to rapid degradation of the clip without any protective measures. Although there was a solid layer of white degradation products on the surface in Fig. 3b, the coated group with a smooth surface and no detachment of corrosion products exhibited better corrosion than the uncoated group. As shown in Fig. 3c, small corrosion pits were observed on the surface, but the MAO/PA composite coating remained intact. This explains why the dual-coated group exhibited better corrosion properties than the other two groups.

### *In vitro* biocompatibility properties

#### CCK-8

**Fig. 4:**
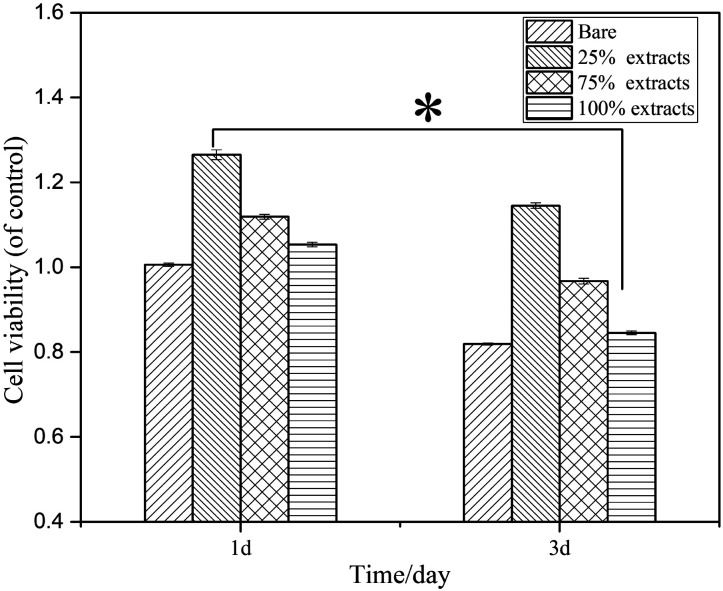
Relative survival rate of human fibroblasts cultured in the bare and MAO/PA-coated magnesium alloy extracts for 1 and 3 d (*P < 0.05).

**Fig. 5:**
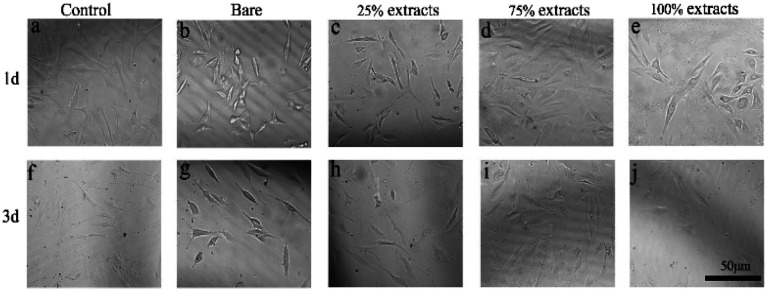
Morphology of fibroblasts cultured in different extract concentrations: (a and f) negative control (DMEM medium containing 20% fetal bovine serum), (b and g) bare extracts, (c and h) 25% extracts, (d and i) 75% extracts, and (e and j) 100% extracts.

Fig. 4 shows that the cell viability of the control group declined gradually with the increase in the MAO/P extract concentration between 1 d and 3 d (*P < 0.05). In addition, the value of the bare group was similar to that of the 100% extract. Overall, the relative cell survival rate was above 75%. Light microscopy revealed that the morphology of the cells was normal, *i.e.*, spindle-shaped (Fig. 5).

#### Flow cytometry apoptosis test

**Fig. 6:**
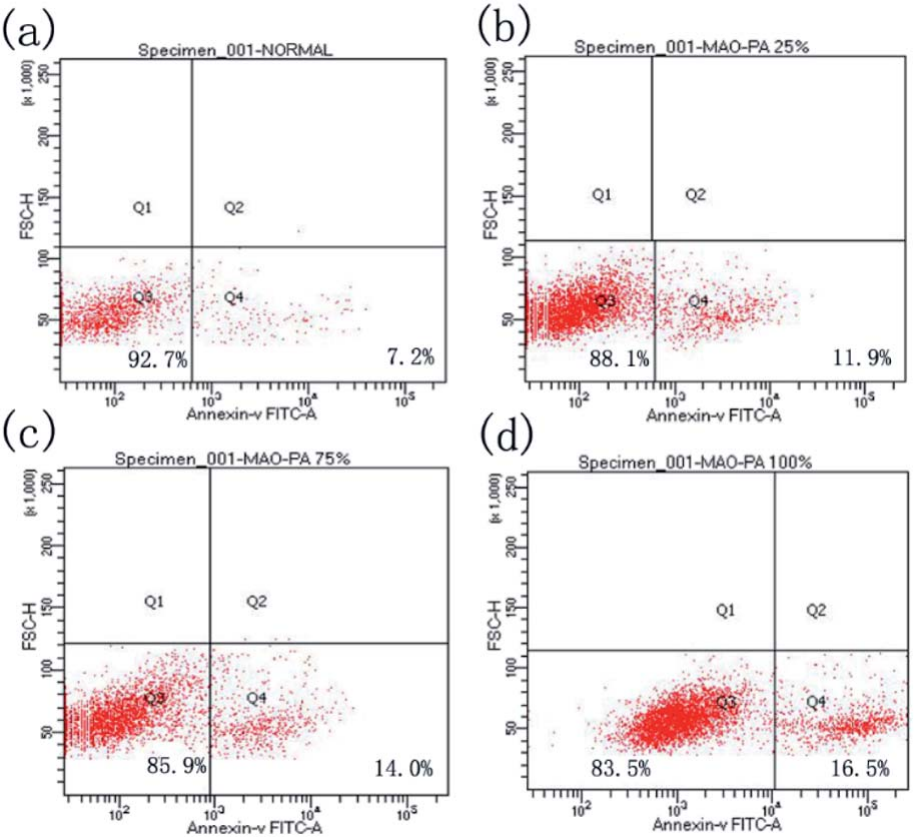
Fluorescence intensity of human fibroblasts cultured in different extracts for 1 d: (a) negative control group; (b) 25% extract; (c) 75% extract; (d) 100% extract. Normal cells are located in the Q3 quadrant, and early apoptotic cells are in the Q4 quadrant.

Apoptotic cells were separated from normal cells by adding Annexin V-FITC. Fig. 6 depicts the fluorescence intensity in human fibroblast cells treated with the control, 25% extract, 75% extract, or 100% extract. The apoptosis rates of human fibroblasts treated with 100% extract, 75% extract, 25% extract, and the control group were 16.5%, 16.5%, 14%, and 7.2%, respectively (Table 2). These results indicated that the apoptosis levels of fibroblasts increased slightly as the extract concentration increased. Some components in the extract of degradable magnesium alloys presumably promote the apoptosis of human fibroblasts.

**Table 2:** Apoptosis rate of human fibroblasts cultured in different extracts for 1 day

Groups	Q3	Q4
Negative control	92.7%	7.2%
25%	88.1%	11.9%
75%	85.9%	14.0%
100%	83.5%	16.5%

#### Cell adhesion

**Fig. 7:**
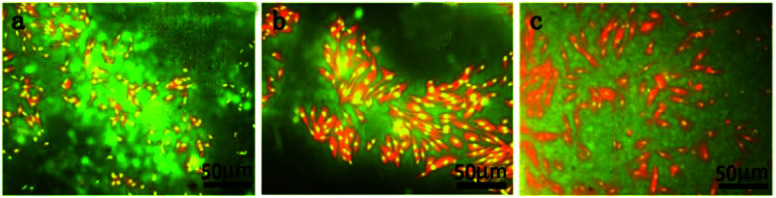
Morphology of human fibroblasts on the membranes at 1 d after culturing on disks with different surface treatment methods: (a) uncoated; (b) PA coated; (c) MAO/PA coated.

Acridine orange (3,6-bis(dimethylamino)acridine hydrochloride) staining was used to observe the cell morphology and analyse cell adhesion based on morphology, which can penetrate the cell membrane after being stained. The cell nucleus is observed as green or yellow green using fluorescence microscopy, but the cytoplasm is red. The weakening or disappearance of fluorescence indicates that the cell is dead. Fig. 7 shows that the morphology of human fibroblasts was normal and spindle-shaped. The outline of the nucleus and cytoplasm was clear, and there was no obvious deformity or apoptosis. This indicated that the uncoated, PA-coated, and MAO/PA-coated magnesium alloy materials were conducive to cell adhesion and exhibited good biocompatibility.

### *In vivo* biocompatibility properties

#### Biochemical results

**Fig. 8:**
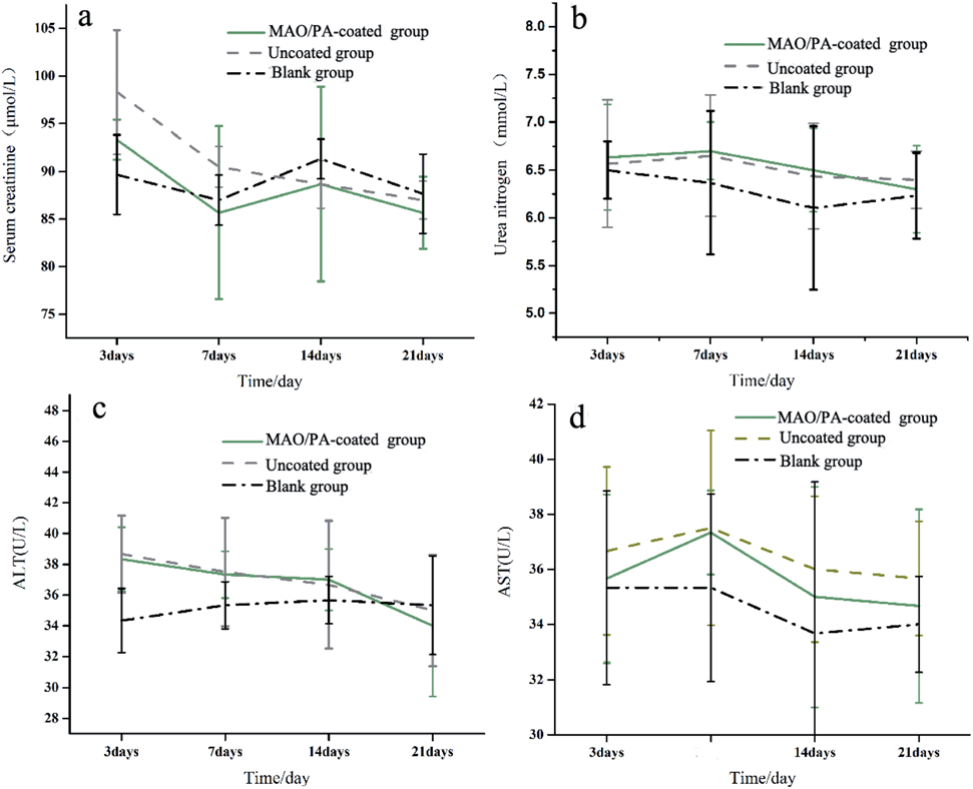
Variation in (a) serum creatinine, (b) serum urea nitrogen, (c) ALT, and (d) AST at 3, 7, 14, and 21 days after operation.

The blood biochemical values (CREA, BUN, ALT, and AST) achieved after implantation of the MAO/PA-coated, uncoated, and blank groups are shown in Fig. 8. The biochemical results in the different groups exhibited no significant difference (*P* > 0.05) and were within the normal range. The BUN, ALT, and AST parameters of the uncoated and MAO/PA-coated groups were slightly higher than those of the blank group at 3, 7, and 14 d after the surgery, but returned to almost similar levels 21 d after the surgery. There was no significant difference in the MAO/PA-coated, uncoated, and blank groups (*P* > 0.05).

#### HE staining assessment

**Fig. 9:**
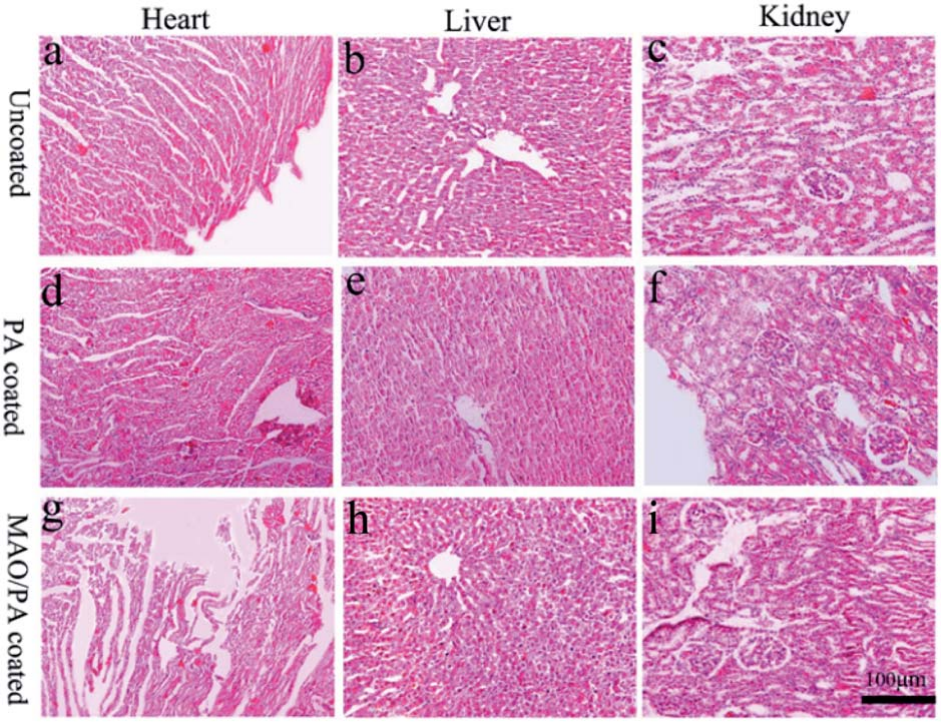
Histological images of the heart, liver and kidney sections three weeks post-operation: (a–c) uncoated group; (d–f) PA coated group; (g–i) MAO/PA-coated group.

Fig. 9 depicts images of the histological heart, liver, and kidney tissues at three weeks. There were no significant signs of inflammation or necrosis in myocardial cells and tissues (Fig. 9a, d, and g). Fig. 9b, e, and h reveals normal hepatic cells, without cell damage, oedema, or degeneration. There was no evident abnormality in the kidneys, as shown in Fig. 9c, f, and i. Overall, the histological images revealed that this type of material had no severe side effects on the essential tissues and organs.

## Discussion

### Degradation behaviour

Electrochemistry is extensively used to determine the instantaneous corrosion performance.^27^ When magnesium alloys react with the SBF solution, the current density represents the kinetics of the corrosion process in the electrolyte, which is an important parameter affecting the corrosion resistance of the magnesium alloy. This is directly related to the corrosion rate; that is, the lower the current density, the better the corrosion resistance.^28^ Thus, the strength of the ongoing corrosion process in a particular electrolyte is revealed.^29^ Although the corrosion potential can also impact the corrosion performance, taking into account the standard deviation of the measured data and the apparent negative value of the established corrosion potential, the general thermodynamic corrosion properties can be negligible.^30^ In this study, the corrosion current of the MAO/PA-coated magnesium alloy decreased by one order of magnitude compared with that of the bare samples (Table 1). One reason is that the MAO technology can form an oxide ceramic coating on the surface of the magnesium alloy.^31^ Another reason is that PA exhibits a strong chelating ability, which can be combined with magnesium ions, zinc ions, and other cations through chemical bonds, thereby reducing the reaction between magnesium ions and corrosion solvents.^32^ Therefore, the MAO/PA-coated magnesium alloy is more corrosion resistant than the bare specimens in SBF. In addition, SEM observations confirmed that the MAO/PA-coated magnesium alloy clips were implanted into rabbits (Fig. 3). However, the corrosion performance must be observed for a long time in clinical applications; at least, it should be degraded stably during the service period to ensure that the clips cannot become detached.

### *In vitro* biocompatibility

According to the ISO 10993-5 standard, a relative cell survival rate above 75% indicates no evident toxicity.^26^ The CCK-8 cytotoxicity test revealed that the MAO/PA-coated magnesium alloy was suitable for biomaterials. Although the morphology of the cells was relatively normal (Fig. 5), the relative survival rate of the cells gradually decreased with increased extract concentration (Fig. 4). Thus, our results demonstrated that high MAO/PA extract concentrations promoted early cell apoptosis in the flow cytometry apoptosis test (Fig. 6 and Table 2). This may be because, as the extract concentration increased, the Mg^2+^ concentration and pH also increased; further, the activation of Mg^2+^ in cells is an early event in cell apoptosis, which is earlier than DNA fragmentation.^33^ Tsao *et al.*^34^ postulated that higher pH values may be related to apoptosis. Ito *et al.*^35^ confirmed that zinc ions released from zinc oxide could cause cytotoxicity when the zinc content in the ceramics exceeds 1.20 wt%, the zinc ions released from zinc oxide could cause cytotoxicity. In addition, it has been found that the element Nd in magnesium alloys can also increase apoptosis.^36^ However, further experimental investigations are required to determine the underlying mechanism. In brief, although the apoptosis rate gradually increased with the extract concentration, the relative survival rate of the cells remained relatively high in a short time, indicating that the MAO/PA-coated magnesium alloy mildly impacted the cells. Therefore, MAO/PA-coated magnesium alloys are promising for use as biomaterials.

The cells are extremely sensitive to changes in the surrounding environment.^37^ However, acridine orange fluorescence staining revealed that the cytoplasm and nucleus of the cells were clear in the early cell adhesion test of the uncoated, PA-coated, and MAO/PA-coated magnesium alloy groups, and the cell morphologies were not significantly abnormal. The results indicated that magnesium alloy supports cell adhesion in the early stages, and the cell compatibility was excellent.

### *In vivo* compatibility

ALT and AST levels are important parameters that can be used to evaluate liver function,^38^ while CREA and BUN are the most widely used parameters to assess renal function; increases in these blood biochemical levels suggest liver and kidney injury.^39^ Furthermore, the main metabolic pathway of magnesium ions is the kidney,^40^ and the liver is the organ that first filters toxins, nutrients, and bacterial metabolites.^41^ Thus, it can be inferred that these two organs are easily damaged when clips are implanted into the body. However, our results suggest that liver and kidney functions were not affected (Fig. 8). Moreover, the results of HE staining confirmed that the clips did not impair the cells and tissues of the heart, liver, and kidney, as shown in Fig. 9. Therefore, the MAO/PA-coated and uncoated Mg–Zn–Y–Nd alloys exhibited good tissue compatibility.

In this study, the MAO/PA-coated magnesium alloy clip exhibits potential feasibility in cholecystectomy due to its good biocompatibility and corrosion resistance. However, there are several limitations: the short experimental period (three weeks) and mechanical properties, which are the major factors used in clinical applications, require further investigation. Therefore, it is necessary to extend the experimental period and further investigate the mechanical properties of the clips in future experiments.

## Conclusions

In this study, *in vitro* and *in vivo* tests were used to assess the corrosion resistance and biocompatibility properties of MAO/PA-coated Mg–Zn–Y–Nd alloys. In the *in vitro* experiments, the MAO/PA coatings significantly improved the corrosion resistance of the magnesium alloy and reduced the corrosion current by approximately one order of magnitude, indicating that the MAO/PA-coated groups had better corrosion properties than the uncoated samples in the rabbits at three weeks. However, the long-term degradation performance requires further investigation. The apoptosis rate of the MAO/PA-coated group increased with increased extract concentration. Further, the cell survival rate satisfied the requirements of biomaterials and exhibited excellent cell adhesion. Moreover, the MAO/PA-coated alloy clips were implanted into rabbits without significant tissue damage or signs of severe foreign body inflammation. Overall, the MAO/PA-coated Mg–Zn–Y–Nd alloy is a safe and biocompatible surgical clip, and there are unlimited possibilities for future clinical applications.

## Conflicts of interest

There are no conflicts to declare.

## Supplementary Material
